# Alteration of Vestibular Function in Pediatric Cochlear Implant Recipients

**DOI:** 10.3389/fneur.2021.661302

**Published:** 2021-05-28

**Authors:** Hajime Koyama, Akinori Kashio, Chisato Fujimoto, Tsukasa Uranaka, Yu Matsumoto, Teru Kamogashira, Makoto Kinoshita, Shinichi Iwasaki, Tatsuya Yamasoba

**Affiliations:** ^1^Department of Otolaryngology and Head and Neck Surgery, Graduate School of Medicine, The University of Tokyo, Tokyo, Japan; ^2^Department of Otolaryngology and Head and Neck Surgery, Graduate School of Medical Sciences and Medical School, Nagoya City University, Nagoya, Japan

**Keywords:** cochlear implantation, vestibular dysfunction, caloric test, damped rotation test, perimodiolar electrode

## Abstract

**Background:** Vestibular dysfunction is a complication of cochlear implantation (CI). Reports on the evaluation of vestibular function before and after CI are limited, especially in children. We investigated the effect of CI on vestibular function in pediatric patients.

**Patients and Methods:** We routinely evaluated vestibular function before but not immediately after CI. Therefore, patients who underwent sequential bilateral CI were enrolled in this study. Seventy-three children who underwent sequential CI from 2003 to 2020 at our hospital were included. Since the vestibular function of the first implanted ear was evaluated before the second surgery for the contralateral ear, post-CI evaluation timing differed among the cases. The evaluation included a caloric test, a cervical vestibular-evoked myogenic potential (cVEMP) test, and a damped rotation test. The objective variables included the results of these tests, and the explanatory variables included the age at surgery, cause of hearing loss, electrode type, and surgical approach used. The associations of these tests were analyzed.

**Results:** cVEMP was the most affected after CI (36.1%), followed by the caloric test (23.6%), and damped rotation test (7.8%). Cochleostomy was significantly more harmful than a round window (RW) approach or an extended RW approach based on the results of the caloric test (*p* = 0.035) and damped rotation test (*p* = 0.029). Perimodiolar electrodes affected the caloric test results greater than straight electrodes (*p* = 0.041). There were no significant associations among these tests' results.

**Conclusions:** Minimally invasive surgery in children using a round window approach or an extended round window approach with straight electrodes is desirable to preserve vestibular function after CI.

## Introduction

Cochlear implantation (CI) is an effective treatment for severe-to-profound sensorineural hearing loss (SNHL). Bilateral CI has been reported to be beneficial for sound localization and for preverbal communication and language development, particularly in pediatric cases (1). However, CI can cause vestibular dysfunction (2–4). The importance of vestibular preservation has been widely acknowledged because bilateral CI recipients are at an increased risk of bilateral vestibular dysfunction. Previous studies have assessed vestibular function after CI in adults (5, 6) and children (7, 8); however, the incidence of vestibular impairment varies across reports. This variation can be attributed to factors such as patient demographics, differences among electrodes, and insertion approach. Few studies have compared the surgical approach (9, 10) as a risk factor for CI-induced vestibular impairment. Hansel et al. analyzed the risks of cochleostomy in a meta-analysis and found a low incidence of subjective vertigo among children (11), but most of the data in the studies included in their meta-analysis were obtained from adult patients; therefore, the identity of the risk factors for vestibular impairment in children remains unclear. Basic research suggests an age-dependent nature of cochlear damage (12–15), and clarification of the vestibular effects and risk factors after CI is needed not only for adults but particularly for children.

Our hospital routinely performs caloric testing, cervical vestibular-evoked myogenic potential (cVEMP) testing, and damped rotation testing to evaluate vestibular function before CI, even in children. In patients undergoing sequential bilateral CI, identical vestibular tests are performed for both the ears. Using these data, we could compare the vestibular function of the first implanted ear before and after the procedure. This study aimed to estimate the deterioration of vestibular function after CI in pediatric patients who received sequential bilateral CI and identify the risk factors for vestibular dysfunction. By assessing the prognostic factors for CI-induced vestibular dysfunction, we sought to shed light on the best strategy for this procedure in children.

## Materials and Methods

### Participants

A total of 73 children (male: 38, female: 35, mean age: 5 years and 7 months ± 3 years and 6 months) who underwent sequential CI for bilateral, severe-to-profound sensorineural hearing loss from 2003 to 2020 at our hospital were included in this study. The mean age at pre-operative evaluation was 2 years and 8 months (range: 5 months−12 years), and the mean age at post-operative evaluation after the second CI was 5 years and 6 months (range: 1 year 4 months−17 years). The mean interval between the pre-operative and post-operative evaluations was 2 years and 9 months (range: 4 months−13 years).

Informed consent was obtained from the parents of all patients. This study was conducted in compliance with the Declaration of Helsinki. The Institutional Review Board of our institution approved this study (2487).

### Vestibular Function Tests

Patients routinely underwent caloric test, cVEMP test, and damped rotation test on both sides before the CI surgery. Post-operative vestibular function in the first implanted ear was measured before the second CI surgery for the contralateral ear. Therefore, we could compare the vestibular function of the first implanted ear before and after CI surgery, and we could measure the change in vestibular function of the non-implanted side during this time without surgery.

We classified the response in each vestibular function test as positive, weak, and negative. We defined the deterioration of vestibular function when the classification changed from positive to weak, positive to negative, or weak to negative, to clearly assess the adverse effect of surgery. Children who were uncooperative or unable to take the test (nine children in caloric test, 17 children in cVEMP test, and 21 children in damped rotation test) could not be assessed, and their data were excluded from the analysis. Those who showed negative responses both pre-operatively and post-operatively (nine children in caloric test, 20 children in cVEMP test, and one child in damped rotation test) were also excluded from the study because we could not accurately evaluate vestibular function alteration caused by surgery. In total, 55, 36, and 51 children were included in caloric test, cVEMP test, and damped rotation test, respectively.

### Caloric Test

Two milliliters of cold water (0°C) was injected into the ear of the children, with their head turned by the examiner, and held for 20 s; thereafter, the water was drained by turning the subject's head, and electronystagmography was performed. We employed a caloric test using only ice water because it requires less time and, thus, is considered more convenient for children than the conventional method. A previous report also has revealed that this technique is useful compared with the conventional approach (16). The duration of induced nystagmus was set as the outcome parameter. Because it was difficult to ask children to look at the targets, calibration was not performed. We evaluated the result as previously reported (17). The normal limit of duration was defined as the average value minus two standard deviations at each age (53.3 s for <24 months, 54.3 s for 25–36 months, 52.4 s for 49–60 months, 48.1 s for 61–72 months, and 35.1 s for >72 months). A positive response was defined when the duration was equal to or longer than the normal limit. The response was considered negative when nystagmus could not be confirmed when water was injected into the implanted ear. A weak response was defined when the duration was shorter than the normal limit.

### Cervical Vestibular-Evoked Myogenic Potential

cVEMP was performed as described previously (18). In summary, surface electromyographic activity of the sternocleidomastoid muscle in response to short tone bursts of 500 Hz (135 dBSPL, rise/fall time 1 ms, plateau time 2 ms) was recorded, and the positive–negative (p13–n23) complex was assessed. The upper limit of the normal cVEMP asymmetry ratio (AR) was set at 34.0. A positive response was defined when reproducible p13–n23 was found in the implanted ear, and cVEMP AR was normal. A negative response was defined when no reproducible p13–n23 was found. A weak response was defined as when reproducible p13–n23 was found in the implanted ear, and cVEMP AR was greater than the normal upper limit.

### Damped Rotation Test

For this test, the children sat on their parent's lap or directly on a chair. The parent restrained the child's head vertically with their arms and hands. The rotation started at 160° per second, and electronystagmography was recorded for 40 s. The chair rotated instantly from this speed toward the non-CI ear, and rotation speed was reduced to zero in 40 s, which indicates that the nystagmus was recorded until the chair stopped. The number of beats was set as the outcome parameter. Calibration was not performed because of similar reasons as that of the caloric test. We evaluated the response as previously reported (17). The normal limit of the number of beats was defined as the average value minus two standard deviations at each age, as was reported by a previous paper for children up to 6 years old (19). For children older than 6 years, the normal limit of number of beats was set as 23, which was based on the number of per-rotatory nystagmus beats in 15 normal children between the ages of 7 and 9 years (31 ± 3.9 beats) recorded in the previous report (17). A positive response was defined when the number of beats was equal to or greater than the normal limit. A response was considered negative when nystagmus could not be confirmed in rotations when the implanted ear was predominantly stimulated. A weak response was defined when the number of beats was less than the normal limit.

### Statistical Analysis

Vestibular function deterioration was defined when the patients had a positive or weak response before surgery that dropped to a weak response or no response after surgery in each vestibular test. Correlations between the different test results (caloric test and cVEMP test, caloric test and damped rotation chair test, and cVEMP test and damped rotation chair test) were estimated. The deterioration of vestibular function was determined as an objective variable, and the causes of hearing loss (genetic or otherwise), age at first CI (younger or older than 2 years), type of electrode (straight-type electrode: CI24M, CI24ST, CI24RST, CI422, CI522; or perimodiolar electrode: CI24RCS, CI24RE, CI512), and insertion approaches [cochleostomy, or extended round window (RW) and RW approaches] were selected as explanatory variables. Age threshold was determined based on reports that cochlear implantation under the age of 2 years is more beneficial for the development of receptive and expressive language skills (20, 21), and that there is a growing need to evaluate the risk of the vestibular deterioration after CI in children under 2 years old. We used Fisher's exact test to assess the risk of each independent variable and the differences among each vestibular test. Statistical significance was set to *p* < 0.05.

## Results

### Patient Demographics

All patients received CI successively. During the follow-up period, no case had any episode of head injury or major complications such as infections that can cause substantial vestibular functional loss after CI.

Table 1 shows the patients' demographics. Genetic mutation was the most frequent cause for SNHL (31/73, 42.4%), followed by viral infection, and syndrome or inner ear malformation. Other causes were neonatal asphyxia and low birth weight. Children were diagnosed with an unknown cause when we were unable to find any genetic mutations, viral infections, other organ diseases, inner ear malformations, or other events that affected hearing function.

**Table 1:** Patient demographics.

Age at pre-operative evaluation (range)			2 years and 8 months. (5 months−12 years)
Age at post-operative evaluation (range)			5 years and 6 months (1 year and 14 months−17 years)
Side of first CI	Left		17
	Right		56
Etiology	Genetic	*GJB2*	26
		*CDH23*	3
		*OTOF*	1
		*TMPRSS3*	1
	Virus	CMV	9
		Rubella	1
		Mumps	1
	Syndrome	Waardenburg	4
		Usher	1
	Inner ear malformations	CC	1
		IP-1	2
		CH-3	1
		CNC stenosis	1
	Other		2
	Unknown		19
Electrode type	Straight type	CI24M	1
		CI24ST	2
		CI24RST	2
		CI422	8
		CI522	27
	Perimodiolar type	CI24RCS	1
		CI24RE	30
		CI512	1
	Slim modiolar type	CI532	1
Surgical approach	Cochleostomy		35
	Extended RW approach		3
	RW approach		35

*CC, common cavity; CMV, cytomegalovirus; CNC, cochlear nerve canal; CH, cochlear hypoplasia; CI, cochlear implantation; RW, round window*.

In the electrode selection, CI522 was the most common straight-type electrode used for surgery, while CI24RE was the most common perimodiolar-type electrode used.

### Vestibular Function Results

**Figure 1:**
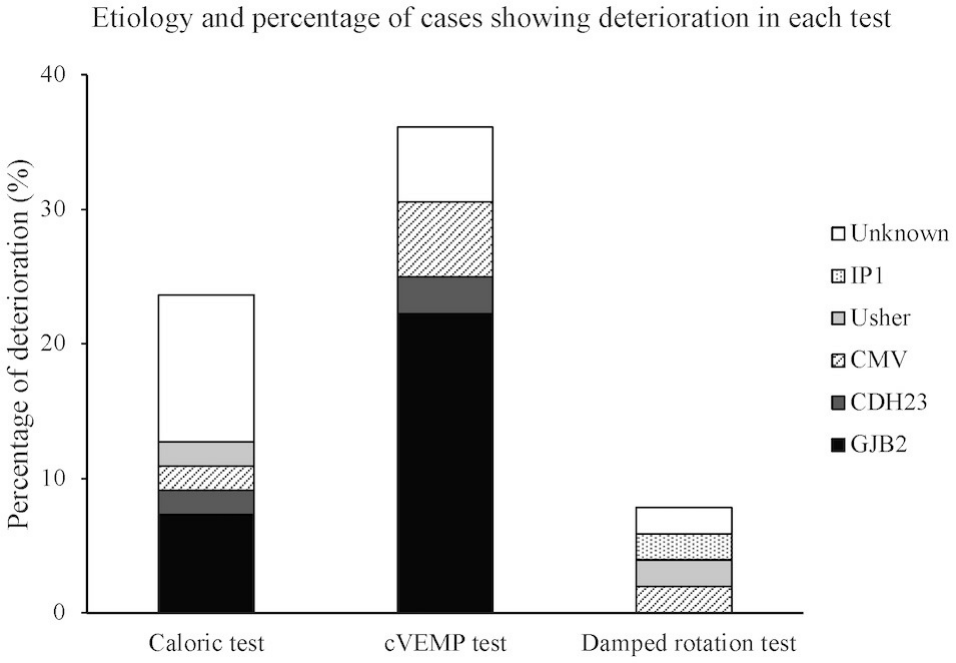
Etiology and percentage of cases showing deterioration in each test. Each bar expresses percentage of deterioration. CMV, cytomegalovirus; IP1, incomplete partition type 1.

The child's vestibular function was considered to deteriorate when the condition changed from positive preoperatively to weak postoperatively, from positive to negative, or from weak to negative in the three vestibular tests. Table 2 shows the number of patients displaying the three patterns of deterioration for each test. The numbers indicate the sum total for each test relative to the total number of children who completed the test. Deterioration of test result was most frequently observed in the cVEMP test (36.1%), followed by the caloric test (23.6%), and then the damped rotation test (7.8%). Figure 1 shows the etiology and percentage of cases that showed deterioration in each test. In the non-CI side, deterioration of test results was observed in only three patients in the caloric test, including one patient who showed the deterioration also in the CI side, four patients in the cVEMP test, including three patients who showed the deterioration in the CI side, and no patient in the damped rotation test.

**Table 2:** Results of vestibular function tests.

**Evaluation change from pre to post**	**Caloric**	**cVEMP**	**Damped rotation chair**
Positive to weak	6	3	4
Positive to negative	5	10	0
Weak to negative	2	0	0
Number of deteriorations	13	13	4
Total number of patients	55	36	51
Percentage of deterioration	23.6%	36.1%	7.8%

*cVEMP, cervical vestibular evoked myogenic potential*.

### Associations of the Different Vestibular Function Test Results

Table 3 shows the associations of the different vestibular test results. Comparison between caloric and cVEMP test results indicates that 31 children underwent both tests. When caloric and damped rotation chair tests were compared, a total of 41 children underwent both tests. Finally, between the cVEMP and damped rotation chair tests, a total of 26 children underwent both tests. However, the *p*-values indicate no significant associations among the three vestibular test results.

**Table 3:** Associations between the vestibular function tests.

		**cVEMP test**		
		**Not deteriorated**	**Deteriorated**	***p*-value**
Caloric test	Not deteriorated	17	8	n.s.
	Deteriorated	4	2	
		**Damped rotation chair test**		
		**Not deteriorated**	**Deteriorated**	***p*****-value**
Caloric test	Not deteriorated	34	2	n.s.
	Deteriorated	3	2	
		**Damped rotation chair test**		
		**Not deteriorated**	**Deteriorated**	***p*****-value**
cVEMP test	Not deteriorated	15	1	n.s.
	Deteriorated	10	0	

*cVEMP, cervical vestibular evoked myogenic potential; n.s., not significant*.

### Risk Factors Analysis

The deterioration of vestibular function based on each test was compared with age at implantation, surgical approach, electrode type inserted, and existence of genetic mutations (Table 4). The number of children categorized with vestibular deterioration, based on each vestibular test result, are shown, and the *p*-values were calculated for comparisons with various parameters. Age and the existence of genetic mutations did not affect any of the vestibular test results. The incidence of reduction in caloric response and damped rotation response was significantly higher (*p* = 0.035 and *p* = 0.029, respectively) among patients in whom an electrode was inserted via a cochleostomy than those who received electrode implantation via a RW or extended RW approach. The patients who received a perimodiolar electrode implantation also showed a significantly higher incidence of reduction in caloric test response (*p* = 0.041) than those who received a straight electrode implantation. However, none of these factors affected the results of the cVEMP test.

**Table 4:** Number of deteriorated and non-deteriorated patients in each test and analysis results.

	**Caloric test**			**cVEMP test**			**Damped rotation test**		
	**Not deteriorated**	**deteriorated**	***p*-value**	**Not deteriorated**	**deteriorated**	***p*-value**	**Not deteriorated**	**deteriorated**	***p*-value**
Age at first CI									
≥2	21	7	n.s.	9	7	n.s.	24	2	n.s.
<2	21	6		14	6		23	2	
Surgical approach									
Cochleostomy	15	9	*p* = 0.035	4	5	n.s.	18	4	*p* = 0.029
RW or ExRW	27	4		19	8		29	0	
Electrode type									
Perimodiolar	15	9	*p* = 0.041	5	5	n.s.	18	3	n.s.
Straight	26	4		17	8		28	1	
Genetic mutation									
Yes	19	5	n.s.	13	9	n.s.	22	0	n.s.
No	23	8		10	4		25	4	

*CI, cochlear implantation; RW, round window; cVEMP, cervical vestibular evoked myogenic potential; n.s., not significant*.

## Discussion

We evaluated the deterioration of vestibular function in children due to CI using caloric test, cVEMP test, and damped rotation test. In the study population, we observed that 23.6% of the tested patients experienced deterioration in caloric test, 36.1% in cVEMP test, and 7.8% in damped rotation test. Reduction in caloric response and damped rotation response was more frequently observed in patients who underwent cochleostomy, and use of a perimodiolar electrode more frequently caused deterioration of caloric response compared with the use of a straight electrode.

Previous studies have demonstrated that CI has a significant negative effect on the results of caloric and cVEMP test results in adults (5) and children (22). A wide range of rates of negative effects has been reported in caloric test (0–30%) (23, 24) and cVEMP test (20–100%) (25–27), and cochleostomy has also been identified as a risk factor for loss of vestibular function in adults (9). Our results were consistent with those of previous reports, and we also clarified that cochleostomy was a risk factor for vestibular dysfunction in children. We also demonstrated that using a straight electrode reduced the incidence of canal damage.

Several mechanisms have been suggested for vestibular dysfunction due to CI, including traumatic injury, fibrosis, electrical stimulation of the otolithic organs, perilymph fluid leakage, and labyrinthitis due to foreign body reaction (28–30). Histopathological studies have shown that CI insertion affects vestibular function by inducing fibrosis in the vestibule, saccule membrane distortion, and cochlear and vestibular hydrops (31, 32). Compared with the RW approach, cochleostomy has also been suggested to cause fibro-osseous reaction and scala vestibuli fibrosis, resulting in vestibular endolymphatic hydrops more frequently (33, 34). Considering these findings, the RW approach is less likely to cause vestibular damage.

Selection of an electrode type is also important for avoiding inner ear trauma. A previous study reported that a straight electrode is less traumatic to the inner ear than a perimodiolar electrode, which has a higher incidence of translocation from the scala tympani to the scala vestibuli (35). Another histopathology report suggested that scala vestibuli involvement, because of damage to the osseous spiral lamina or basilar membrane in the cochlear basal turn, was highly correlated with vestibular damage (31). Various reports have demonstrated the straight electrode's superiority in cochlear preservation by estimating residual hearing ability (36–38), and a temporal bone study suggested that use of a straight electrode minimized trauma to the intracochlear structures (39). Our results also suggest that a straight electrode is desirable not only for hearing preservation but also for vestibular preservation.

Previous studies have demonstrated that cVEMP is more frequently affected than caloric test or damped rotation chair test (3, 40, 41), as shown in the current study. The saccule may be more susceptible to insertion damage due to its anatomical proximity to the RW. Another reason cVEMP is more frequently affected could be because the packing following implantation may induce conductive hearing loss (42, 43); however, the fascia placed on the RW for packing is very small and may have a limited effect on conduction efficacy. It is noteworthy that the approach of electrode insertion and the electrode type caused a difference in the incidence of CI-induced negative effect on caloric test and rotation test but not on cVEMP. Our results are compatible with previous studies that showed no significant differences in cVEMP between electrode types (44) and between approaches of electrode insertion (22).

It is important to clarify the correlation between the anatomy of the inner ear and the vestibular testing. However, only four cases of inner ear malformation met the criteria of having preoperative and postoperative vestibular testing, and most of them showed a negative response at preoperative evaluation. Because of this limited number of patients, we could not show the correlation between the anatomy of the inner ear and the vestibular testing.

This study has several limitations. First, some children were excluded from this study because they could not be assessed either preoperatively or postoperatively or showed a negative response in vestibular function tests, both preoperatively and postoperatively. Furthermore, when CI was performed on the negative response side, we were unable to detect vestibular function changes. Second, we could not perform a quantitative assessment for either caloric or damped rotation test because we could not complete calibration with the children either preoperative or postoperative condition. Hence, these results contain variability not only at each point but also between these two time points. Moreover, children are less likely to follow our instructions, which lessens reliability of vestibular test results compared with those of the adults. We attempted to overcome the problem by classifying the test results as previously reported (17, 21). In the current study, deterioration of test results in the non-CI side was observed in only three patients in caloric test, including one who showed the deterioration also in the CI-side, four patients in cVEMP test, including three patients who showed the deterioration in the CI-side, and no patient in damped rotation test. These findings support the high reliability of our test results. Third, electrode type and surgical approach can be correlated, but we were unable to separate the negative effects of these variables. A RW approach is not preferable with a perimodiolar electrode because of anatomical obstacles, namely, the anteroinferior region of the RW bony margin (45). Thus, a straight electrode and RW approach are recommended for vestibular function preservation. As a result, surgeons tend to perform cochleostomy when using a perimodiolar electrode, but an RW approach when using a straight electrode. Fourth, this is a retrospective study, and postoperative evaluation timing differed because the second test was performed just before the second CI, which meant that there was the variety of the intervals. Dysfunctions of the peripheral vestibular receptors could be induced not only by a cochlear implant electrode insertion but also by different mechanisms such as infections or traumatic head injury, and some children with SNHL may experience progressive vestibular dysfunction (46). We could not rule out the effect of these different mechanisms unrelated to the inserted electrode or spontaneous progression of vestibular dysfunction unrelated to CI. In the current study, no patient had experienced head injury after CI or major complications such as infections, which could lessen these effects, and three patients who showed deterioration in the implanted ear showed deterioration of cVEMP in the contralateral non-CI ear, implying that the progressive vestibular dysfunction may not be due to surgery. However, only one patient who showed deterioration on the implanted side showed deterioration on the contralateral side in the caloric test, and no patient showed such deterioration in damped rotation test. A previous report stated that vestibular modifications following the implant were stable (24) and another paper that compared the vestibular test sets performed with different intervals concluded that the differences were not likely a confounder that would influence the results of postoperative decline (10).

In summary, we assessed vestibular function using caloric test, cVEMP test, and damped rotation test before and after CI in children. We evaluated the negative effect of CI on vestibular function based on causes of hearing loss, age at first CI, type of electrode used, and insertion approaches, and concluded that cochleostomy and use of a perimodiolar electrode are risk factors for postoperative deterioration of vestibular function. Although CI is beneficial for children with SNHL, a straight electrode with an RW or extended RW approach would be preferable to preserve vestibular function. Similar results have already been reported in adults, but there were limited number of studies evaluating children. The current study confirms that these findings are also applicable in children. Children with SNHL tend to receive bilateral CI due to bilateral hearing benefit, and the need for preservation of vestibular function is increasing. Therefore, an RW or an extended RW approach with straight electrodes is desirable to preserve vestibular function after CI, especially for children.

## Data Availability Statement

The original contributions presented in the study are included in the article/supplementary material, further inquiries can be directed to the corresponding author.

## Ethics Statement

The studies involving human participants were reviewed and approved by Graduate School of Medicine and Faculty of Medicine, The University of Tokyo, Research Ethics Committee. Written informed consent to participate in this study was provided by the participants' legal guardian/next of kin.

## Author Contributions

HK, AK, CF, and TY contributed to conception and design of the study. HK organized the database, performed the statistical analysis, and wrote the first draft of the manuscript. AK and CF wrote sections of the manuscript. TU, YM, TK, MK, SI, and TY revising it critically for important intellectual content. All authors contributed to manuscript revision, read, and approved the submitted version.

## Conflict of Interest

The authors declare that the research was conducted in the absence of any commercial or financial relationships that could be construed as a potential conflict of interest.

## References

[1] Lammers MJW van der Heijden GJMG Pourier VEC Grolman W. Bilateral cochlear implantation in children: a systematic review and best-evidence synthesis. Laryngoscope. (2014) 124:1694–9. 10.1002/lary.24582 24390811

[2] Brey RH Facer GW Trine MB Lynn SG Peterson AM Suman VJ. Vestibular effects associated with implantation of a multiple channel cochlear prosthesis. Am J Otol. (1995) 16:424–30. 8588641

[3] Buchman CA Joy J Hodges A Telischi FF Balkany TJ. Vestibular effects of cochlear implantation. Laryngoscope. (2004) 114:1–22. 10.1097/00005537-200410001-00001 15454752

[4] Chiesa Estomba CM Rivera Schmitz T Betances Reinoso FA Dominguez Collado L Estevez Garcia M Lorenzo AI. Complications after cochlear implantation in adult patients. 10-year retrospective analysis of a tertiary academic centre. Auris Nasus Larynx. (2017) 44:40–5. 10.1016/j.anl.2016.03.012 27146006

[5] Ibrahim I Da Silva SD Segal B Zeitouni A. Effect of cochlear implant surgery on vestibular function: meta-analysis study. J Otolaryngol Head Neck Surg. (2017) 46:44. 10.1186/s40463-017-0224-0 28595652 PMC5465585

[6] Colin V Bertholon P Roy S Karkas A. Impact of cochlear implantation on peripheral vestibular function in adults. Eur Ann Otorhinolaryngol Head Neck Dis. (2018) 135:417–20. 10.1016/j.anorl.2018.10.007 30431000

[7] Yong M Young E Lea J Foggin H Zaia E Kozak FK. Subjective and objective vestibular changes that occur following paediatric cochlear implantation: systematic review and meta-analysis. J Otolaryngol Head Neck Surg. (2019) 48:22. 10.1186/s40463-019-0341-z 31118089 PMC6530180

[8] Talaat HS Chedid AIF Wageih GM Zein El-Abedein AM. Vestibular function assessment following cochlear implantation using rotatory chair testing. Eur Arch Oto-Rhino-Laryngol. (2020). 10.1007/s00405-020-06308-w. [Epub ahead of print]. 32857183

[9] Todt I Basta D Ernst A. Does the surgical approach in cochlear implantation influence the occurrence of postoperative vertigo? Otolaryngol Head Neck Surg. (2008) 138:8–12. 10.1016/j.otohns.2007.09.003 18164986

[10] Frodlund J Harder H Mäki-Torkko E Ledin T. Vestibular function after cochlear implantation: a comparison of three types of electrodes. Otol Neurotol. (2016) 37:1535–40. 10.1097/MAO.0000000000001229 27749755

[11] Hansel T Gauger U Bernhard N Behzadi N Romo Ventura ME Hofmann V. Meta-analysis of subjective complaints of vertigo and vestibular tests after cochlear implantation. Laryngoscope. (2018) 128:2110–23. 10.1002/lary.27071 29314057

[12] Campo P Pouyatos B Lataye R Morel G. Is the aged rat ear more susceptible to noise or styrene damage than the young ear? Noise Health. (2003) 5:1–18. 12804208

[13] Boettcher FA. Susceptibility to acoustic trauma in young and aged gerbils. J Acoust Soc Am. (2002) 112:2948–55. 10.1121/1.1513364 12509015

[14] Palomar García V Abdulghani Martínez F Bodet Agustí E Andreu Mencía L Palomar Asenjo V. Drug-induced toxicity: current status. Acta Otolaryngol. (2001) 121:569–72. 10.1080/00016480121545 11583387

[15] Khan HA Al Deeb S Al Moutaery K Tariq M. Influence of age on iminodipropionitrile-induced vestibular and neurobehavioral toxicities in rats. Exp Toxicol Pathol. (2003) 55:181–6. 10.1078/0940-2993-00312 14620540

[16] Schmäl F Lübben B Weiberg K Stoll W. The minimal ice water caloric test compared with established vestibular caloric test procedures. J Vestib Res. (2005) 15:215–22. 16286703

[17] Inoue A Iwasaki S Ushio M Chihara Y Fujimoto C Egami N. Effect of vestibular dysfunction on the development of gross motor function in children with profound hearing loss. Audiol Neurotol. (2013) 18:143–51. 10.1159/000346344 23392310

[18] Fujimoto C Suzuki S Kinoshita M Egami N Sugasawa K Iwasaki S. Clinical features of otolith organ-specific vestibular dysfunction. Clin Neurophysiol. (2018) 129:238–45. 10.1016/j.clinph.2017.11.006 29207275

[19] Kaga K Suzuki JI Marsh RR Tanaka Y. Influence of labyrinthine hypoactivity on gross motor development of infants. Ann NY Acad Sci. (1981) 374:412–20. 10.1111/j.1749-6632.1981.tb30887.x 6978638

[20] Anderson I Weichbold V D'Haese PSC Szuchnik J Quevedo MS Martin J. Cochlear implantation in children under the age of two - What do the outcomes show us? Int J Pediatr Otorhinolaryngol. (2004) 68:425–31. 10.1016/j.ijporl.2003.11.013 15013608

[21] Govaerts PJ De Beukelaer C Daemers K De Ceulaer G Yperman M Somers T. Outcome of cochlear implantation at different ages from 0 to 6 years. Otol Neurotol. (2002) 23:885–90. 10.1097/00129492-200211000-00013 12438851

[22] Cozma RS Hera MC Cobzeanu MD Olariu R Bitere OR Mârtu C. Saccular function evolution related to cochlear implantation in hearing impaired children. Rom J Morphol Embryol. (2020) 1:113–9. 10.47162/RJME.61.1.12 32747901 PMC7728102

[23] Ajalloueyan M Saeedi M Sadeghi M Zamiri Abdollahi F. The effects of cochlear implantation on vestibular function in 1-4 years old children. Int J Pediatr Otorhinolaryngol. (2017) 94:100–3. 10.1016/j.ijporl.2017.01.019 28166997

[24] Jacot E Van Den Abbeele T Debre HR Wiener-Vacher SR. Vestibular impairments pre- and post-cochlear implant in children. Int J Pediatr Otorhinolaryngol. (2009) 73:209–17. 10.1016/j.ijporl.2008.10.024 19101044

[25] Moushey-Bogle J. The effect of Cochlear implantation on the vestibular evoked myogenic potential response in children and adults. Speech, Language, and Hearing Services Graduate Theses and Dissertations (2010).

[26] Jin Y Nakamura M Shinjo Y Kaga K. Vestibular-evoked myogenic potentials in cochlear implant children. Acta Otolaryngol. (2006) 126:164–9. 10.1080/00016480500312562 16428194

[27] Psillas G Pavlidou A Lefkidis N Vital I Markou K Triaridis S. Vestibular evoked myogenic potentials in children after cochlear implantation. Auris Nasus Larynx. (2014) 41:432–5. 10.1016/j.anl.2014.05.008 24882586

[28] Licameli G Zhou G Kenna MA. Disturbance of vestibular function attributable to cochlear implantation in children. Laryngoscope. (2009) 119:740–5. 10.1002/lary.20121 19205016

[29] Bance ML O'Driscoll M Giles E Ramsden RT. Vestibular stimulation by multichannel cochlear implants. Laryngoscope. (1998) 108:291–4. 10.1097/00005537-199802000-00025 9473085

[30] O'Leary MJ Fayad J House WF Linthicum FH. Electrode insertion trauma in cochlear implantation. Ann Otol Rhinol Laryngol. (1991) 100:695–9. 10.1177/000348949110000901 1952658

[31] Tien HC Linthicum FH. Histopathologic changes in the vestibule after cochlear implantation. Otolaryngol Head Neck Surg. (2002) 127:260–4. 10.1067/mhn.2002.128555 12402002

[32] Handzel O Burgess BJ Nadol JB. Histopathology of the peripheral vestibular system after cochlear implantation in the human. Otol Neurotol. (2006) 27:57–64. 10.1097/01.mao.0000188658.36327.8f 16371848

[33] Ishiyama A Doherty J Ishiyama G Quesnel AM Lopez I Linthicum FH. Post hybrid cochlear implant hearing loss and endolymphatic hydrops. Otol Neurotol. (2016) 37:1516–21. 10.1097/MAO.0000000000001199 27608418 PMC5102757

[34] Su-Velez BM Lopez IA Ishiyama A Ishiyama G. Human temporal bone study of vestibular histopathology in cochlear implant patients with cochlear hydrops. Otol Neurotol. (2020) 41:e607–14. 10.1097/MAO.0000000000002609 32150024 PMC7415216

[35] Dhanasingh A Jolly C. An overview of cochlear implant electrode array designs. Hear Res. (2017) 356:93–103. 10.1016/j.heares.2017.10.005 29102129

[36] Skarzynski H Matusiak M Lorens A Furmanek M Pilka A Skarzynski PH. Preservation of cochlear structures and hearing when using the Nucleus Slim Straight (CI422) electrode in children. J Laryngol Otol. (2016) 130:332–9. 10.1017/S0022215115003436 26763105

[37] Skarzynski H Lorens A Matusiak M Porowski M Skarzynski PH James CJ. Cochlear implantation with the nucleus slim straight electrode in subjects with residual low-frequency hearing. Ear Hear. (2014) 35:e33–43. 10.1097/01.aud.0000444781.15858.f1 24556970

[38] Moran M Dowell RC Iseli C Briggs RJS. Hearing preservation outcomes for 139 cochlear implant recipients using a thin straight electrode array. Otol Neurotol. (2017) 38:678–84. 10.1097/MAO.0000000000001374 28353622

[39] Lenarz T Stöver T Buechner A Paasche G Briggs R Risi F. Temporal bone results and hearing preservation with a new straight electrode. Audiol Neurotol. (2006) 11:34–41. 10.1159/000095612 17063009

[40] Basta D Todt I Goepel F Ernst A. Loss of saccular function after cochlear implantation: the diagnostic impact of intracochlear electrically elicited vestibular evoked myogenic potentials. Audiol Neurotol. (2008) 13:187–92. 10.1159/000113509 18212494

[41] Krause E Louza JPR Wechtenbruch J Gürkov R. Influence of cochlear implantation on peripheral vestibular receptor function. Otolaryngol Head Neck Surg. (2010) 142:809–13. 10.1016/j.otohns.2010.01.017 20493350

[42] Melvin TA Della Santina CC Carey JP Migliaccio AA. The effects of cochlear implantation on vestibular function. Otol Neurotol. (2009) 30:87–94. 10.1097/MAO.0b013e31818d1cba 19108038 PMC2767271

[43] Merchant GR Schulz KM Patterson JN Fitzpatrick D Janky KL. Effect of cochlear implantation on vestibular evoked myogenic potentials and wideband acoustic immittance. Ear Hear. (2020) 41:1111–24. 10.1097/AUD.0000000000000831 32032225 PMC7392788

[44] Imai T Okamura T Ohta Y Oshima K Sato T Kamakura T. Effects of cochlear implants on otolith function as evaluated by vestibulo-ocular reflex and vestibular evoked myogenic potentials. Auris Nasus Larynx. (2019) 46:836–43. 10.1016/j.anl.2019.03.011 31010711

[45] Souter MA Briggs RJS Wright CG Roland PS. Round window insertion of precurved perimodiolar electrode arrays: how successful is it? Otol Neurotol. (2011) 32:58–63. 10.1097/MAO.0b013e3182009f52 21131883

[46] Bernard S Wiener-Vacher S Van Den Abbeele T Teissier N. Vestibular disorders in children with congenital cytomegalovirus infection. Pediatrics. (2015) 136:e887–95. 10.1542/peds.2015-0908 26347442

